# Role of cardiovascular magnetic resonance in suspected cardiac amyloidosis: late gadolinium enhancement pattern as mortality predictor

**DOI:** 10.1007/s12471-017-1046-4

**Published:** 2017-10-20

**Authors:** M. Baroni, S. Nava, G. Quattrocchi, A. Milazzo, C. Giannattasio, A. Roghi, P. Pedrotti

**Affiliations:** 1 Cardiologia 3, A. De’ Gasperis Heart Center, ASST Grande Ospedale Metropolitano Niguarda, Milano, Italy; 2 Cardiologia 1, A. De’ Gasperis Heart Center, ASST Grande Ospedale Metropolitano Niguarda, Milano, Italy; 3 Cardiologia 4, A. De’ Gasperis Heart Center, ASST Grande Ospedale Metropolitano Niguarda, Milano, Italy; 4 Health Science Department, Bicocca University, Milano, Italy

**Keywords:** Amyloidosis, Cardiac magnetic resonance

## Abstract

**Background:**

Cardiac magnetic resonance (CMR) has gained a central role in the diagnosis of cardiac amyloidosis (CA). While the diagnostic role of a typical late gadolinium enhancement (LGE) pattern (global subendocardial enhancement coupled with accelerated contrast washout) has been identified, evidence is still conflicting regarding the prognostic role of such examination.

**Methods and results:**

We retrospectively analysed all patients referring for CMR at Niguarda Hospital (Milan, Italy) from January 2006 to January 2015 for suspected CA. Primary outcome was all-cause mortality. We identified 42 patients and divided them into 2 groups, according to the presence (Group A) or absence (Group B) of a typical amyloidosis LGE pattern. At the end of the follow-up (median 37 months, interquartile range 10–50 months), 31 patients (74%) had died. The hazard ratio for all-cause death was 3.2 (95% confidence interval [CI] 1.5–6.4, *p* < 0.01) for Group A versus Group B. Median survival time was 17 months (95% CI 7–42 months) for Group A and 70 months (95% CI 49–94 months) for Group B (*p* < 0.01). Multivariate analysis did not find any adjunctive predictive role for biventricular volumes and ejection fraction, indexed left ventricular mass, transmitral E/e’ at echocardiography, age at diagnosis or serum creatinine.

**Conclusion:**

In our population, a typical LGE pattern was significantly associated with higher mortality. Moreover, patients with a typical LGE pattern showed a globally worse prognosis. Our data suggest that the LGE pattern may play a central role in prognostic stratification of patients with suspected CA, thus prompting further diagnostic and therapeutic measures.

## Background

Infiltrative cardiomyopathies are a wide spectrum of rare cardiac diseases characterised by chronic deposition of pathological substance into cardiac muscle, thus causing its progressive stiffening, diastolic heart failure and an overall poor outcome. Amyloidosis, caused by a misfolded, insoluble aggregated protein featuring a characteristic beta-sheet structure, is the most common aetiology [1]. While different amyloid proteins have been described over the years, only two forms of cardiac amyloidosis (CA) account for the majority of cases. AL amyloidosis, caused by immunoglobulin light chains produced by a mutated plasma cell clone, is the most common form accounting for about two thirds of cases of CA. Transthyretin-related (ATTR) amyloidosis can reveal itself as an inherited or a sporadic (senile systemic) form, with a variable range of severity [2]. Over the past few years, cardiac magnetic resonance (CMR) has gained a central role in the diagnostic workup of infiltrative cardiomyopathies. Allowing tissue characterisation, CMR has proven to be a key diagnostic tool when routine cardiac echography and the patient’s history are suggestive of CA. A typical pattern of late gadolinium enhancement (LGE) has been described in patients with CA, characterised by global subendocardial enhancement with accelerated gadolinium washout [3].

While the diagnostic accuracy of CMR has been highlighted by several studies, evidence is still conflicting about the prognostic role of this type of examination for patients with suspected CA. This study aimed to assess the prognostic value of CMR in a group of consecutive patients routinely referred for a CMR scan in the work-up of suspected CA in a tertiary referral centre.

## Methods

### Population

We searched the database of the CMR Laboratory of Niguarda Hospital (Milan, Italy) from January 2006 to January 2012 for patients referred for suspected CA. We included patients with known systemic amyloidosis (ATTR or AL), as well as patients referred for a suspected infiltrative disorder at clinical and echocardiographic assessment, with evidence of congestive heart failure and diastolic dysfunction or myocardial hypertrophy with a discordance between electrocardiogram (ECG) QRS voltage and left ventricular wall thickness. We accurately excluded patients with evidence of increase of left ventricular mass due to valvular diseases, hypertension or hypertrophic cardiomyopathy.

### Sources of data

Baseline clinical, laboratory and echocardiographic data were obtained from internal medical records. When a parameter was repeatedly available, only the closest to the time of CMR was considered.

Echocardiographic data included left ventricular volumes, left ventricular mass (Devereux formula) and ejection fraction, as well as Doppler markers for diastolic dysfunction (mitral E/e’ ratio). Laboratory markers included blood cell count, renal function and *N*-terminal pro-brain natriuretic peptide (NT-proBNP).

### CMR Protocol

Patients were scheduled for contrast-enhanced CMR after the exclusion of contraindications to CMR and gadolinium-based contrast agents. Scans were performed on a 1.5 T clinical scanner (Siemens Avanto, Erlangen, Germany) using a four-element phased-array receiver coil. All images were acquired with retrospective ECG gating and during repeated single breath-holds. Balanced steady state free precession cine images (echo time/repetition time, 1.6/3.2 ms; flip angle, 60°; typical pixel size, 2.4 × 1.4 mm) were acquired in three long-axis planes and in contiguous short-axis slices (slice thickness, 8 mm; gap, 2 mm) from the atrioventricular groove to the apex. LGE images were acquired 5 and 10 minutes after a 0.1 mmol/kg infusion of gadopentetate dimeglumine (Bayer AG Magnevist, Leverkusen, Germany) on planes matching cine images, with a segmented inversion-recovery gradient echo sequence (echo time/repetition time, 1.4/5.4 ms; flip angle, 10°; slice thickness, 8 mm; gap, 2 mm; typical spatial resolution, 2.2 × 1.5 mm; inversion times adjusted to null normal myocardium), after inversion time identification with a scout acquisition [4]. Considering the technical challenge to correctly null the myocardium in CA patients, also phase-sensitive inversion recovery (PSIR) sequences were acquired.

**Fig. 1:**
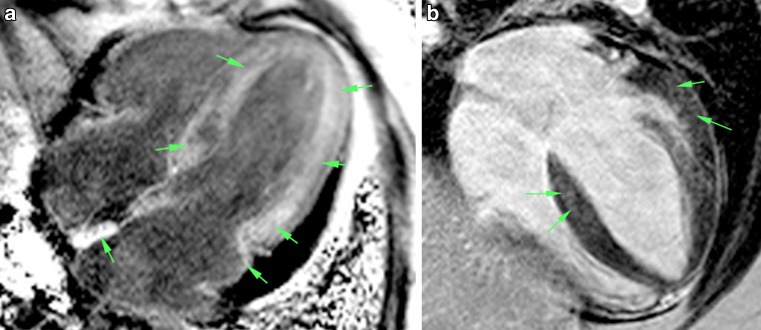
Typical (**a**) and atypical (**b**) LGE patterns. Both images are acquired with PSIR sequence in 4‑chamber long axis view. Areas of LGE are indicated by *arrows*. *LGE* late gadolinium enhancement, *PSIR* phase-sensitive inversion recovery

**Fig. 2:**
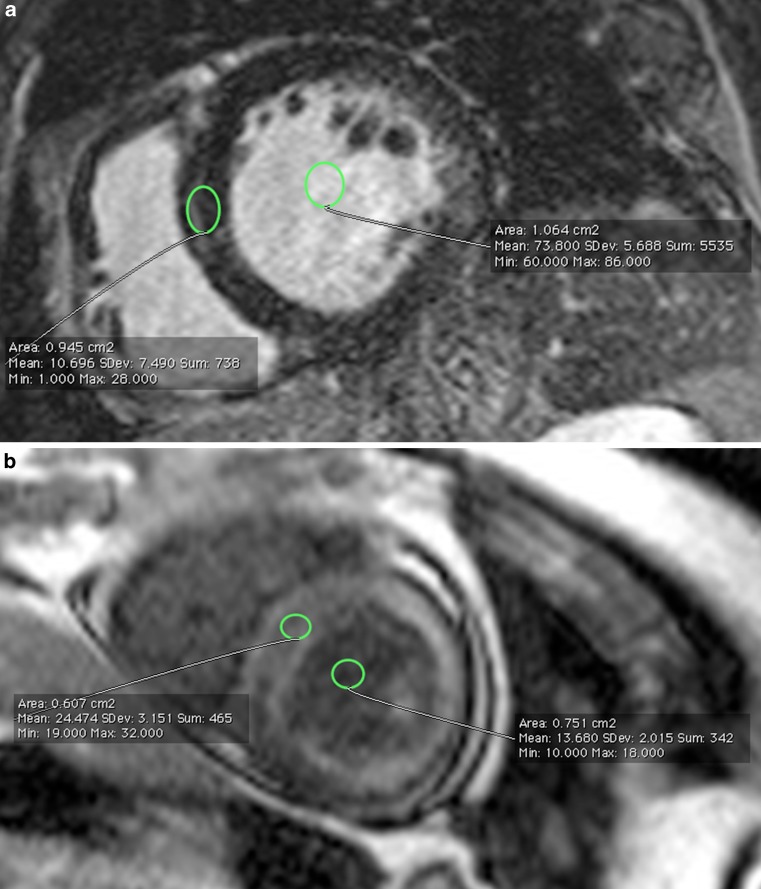
Example of blood/myocardial T1 ratio determination for semi-quantitative description of contrast washout. Both images are acquired with segmented inversion-recovery gradient echo sequence. **a** accelerated contrast washout. Blood T1 = 13 ms, myocardial T1 = 24 ms, blood/myocardial ratio = 0.5. **b** normal contrast washout. Blood T1 = 60 ms, myocardial T1 = 10 ms, blood/myocardial ratio = 6

Image analysis was performed by 2 experienced observers (PP, AR) blinded to patient identity on a commercially available cardiovascular magnetic resonance workstation (Leonardo, Siemens, Erlangen, Germany). Ventricular volumes and myocardial mass were quantified from short-axis cine images by standard methods [5] and indexed to body surface area. The observers independently determined the dichotomous presence or absence of myocardium LGE by reviewing all post-contrast CMR images; discordant interpretations were resolved by consensus. LGE distribution was classified as: typical LGE pattern, with global subendocardial enhancement with or without any transmural enhancement and atypical LGE pattern, with any other non-ischaemic pattern (Fig. 1). Due to the lack of a standardised method for quantitative description of washout kinesis, this parameter was assessed by the ratio of blood and myocardial T1 values, measured 5 minutes after gadolinium infusion. Myocardial T1 was measured on the left ventricular subendocardium of the interventricular septum while blood T1 was measured in the middle of the left ventricular chamber (Fig. 2) in a mid-ventricular short-axis slice. A blood/myocardium value <1 (i. e. more gadolinium in the myocardium than in the blood 5 minutes after injection) was considered a marker of accelerated contrast washout.

### Endomyocardial biopsy and genetic analysis

Patients were referred to endomyocardial biopsy or genetic analysis at the discretion of the treating physician. Endomyocardial biopsy was performed via the internal jugular vein or the femoral vein with a standard bioptome. At least three samples were taken from the right ventricular apex and interventricular septum and processed at the pathologist’s discretion.

For genetic analysis, DNA was extracted from whole blood and amplified by polymerase chain reaction assay, and the coding region of the TTR gene was sequenced looking for most common spot mutations.

### Study endpoint

Primary endpoint was all-cause mortality. Data was obtained from patients’ records and from the national health service’s electronic archives. Death status was determined as of month and year. The most recent date of the clinical follow-up was recorded for survived patients.

### Statistical analysis

Statistical analysis was performed with Med Calc v.14 (Medcalc Software, Ostend, Belgium) and SPSS v.22 software (IBM corp, Armonk, USA). Continuous variables were tested for normality with the Shapiro-Wilk test. Normal variables are presented as mean ± standard deviation (SD), otherwise as median and interquartile range (IQR). Differences in the mean values were compared using a two-tailed *t*-test for continuous paired and unpaired variables and a Chi-square test for nominal values. A *p*-value <0.05 was considered statistically significant. Survival was evaluated with a Cox proportional hazards regression analysis, providing hazard ratios with 95% confidence intervals and Kaplan-Meier curves. Variables selected for clinical relevance were first explored with the univariate Cox regression model and then entered into the multivariate model.

## Results

**Fig. 3:**
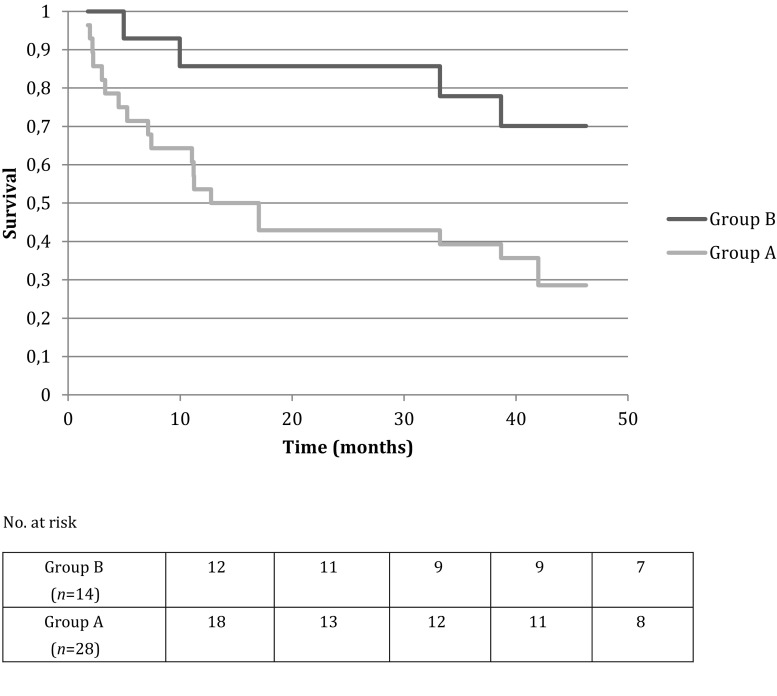
Kaplan Meyer survival curve. *p* < 0.01 at Log Rank test

Of 7,800 CMR scans performed in the aforementioned period, 42 patients (31 males, mean age at CMR 58 ± 12 years) were included for analysis. Patients were divided into Group A and Group B, based on LGE pattern analysis. Patients in Group A (28; 67%) had a typical LGE pattern, as previously defined, while patients in Group B (14; 33%) had an atypical LGE pattern. A detailed description of the diagnostic findings is summarised in Tab. 1. Patients in Group A had more frequently LGE of atrial walls and an accelerated contrast washout. There were no significant differences in clinical features nor in other imaging findings between the 2 groups (Tab. 2). After a median follow up of 37 months (IQR 10–50 months), 31 patients (74%) had died. Patients in Group A had an hazard ratio for all-cause death of 3.2 compared to patients in Group B (95% CI 1.5–6.4, *p* < 0.01) (Fig. 3). Median survival time was 17 months (95% CI 7–42 months) for Group A and 70 months (95% CI 49–94 months) for Group B (*p* < 0.01). Biventricular volumes and ejection fraction, indexed left ventricular mass (I-LVM), E/e’ at echo, age at diagnosis and serum creatinine didn’t show any adjunctive predictive value at multivariate analysis (Tab. 3). Endomyocardial biopsy or genetic analysis was performed in 15 patients (54%) of Group A and in 13 patients (93%) of Group B. Diagnosis of amyloidosis was confirmed in 13 patients in Group A (4 mutated ATTR, 1 senile systemic ATTR, 8 AL) and in 1 patient in Group B (1 senile systemic ATTR, *p* < 0.05) (Tab. 1), thus CMR sensitivity and specificity for the diagnosis of CA were 93% and 87%, respectively.

**Table 1:** Diagnostic findings in study groups

		GROUP A (*n* = 28; 67%)	GROUP B (*n* = 14; 33%)
LGE PATTERN	Global subendocardial (*n*; %)	18 (64%)	0
	Global subendocardial, focally or globally transmural (*n*; %)	10 (36%)	0
	Midwall (*n*;%)	0	6 (43%)
	Atrial walls (*n*;%)	25 (89%)	3 (21%)
	Subepicardial, pericardial (*n*;%)	0	2 (14%)
	Accelerated contrast wash-out (*n*;%)	23 (82%)	0
	No LGE (*n*;%)	0	3 (21%)
EMB	Negative (*n*;%)	1 (3%)	10 (71%)
	Positive (*n*;%)	9 (32%) *[8 AL, 1 WT ATTR]*	1 (7%) [WT ATTR]
	NP (*n*;%)	18 (64%)	3 (21%)
GA	Negative (*n*;%)	10 (35%)	6 (42%)
	Positive (*n*;%)	4 (14%)	0
	NP (*n*;%)	14 (50%)	8 (57%)

*LGE* late gadolinium enhancement, *EMB* endomyocardial biopsy, *GA* genetic analysis, *AL* amyloid light chain, *WT* wild type, *ATTR* transthyretin-related,* NP* not performed

**Table 2:** Baseline characteristics of study groups

	GROUP A (*n* = 28; 67%)	GROUP B (*n* = 14; 33%)	
Age at CMR (years)	54 ± 14	64 ± 11	*p* = 0.44
LV EDV (echo) (ml)	43 ± 12	45 ± 14	*p* = 0.65
LV EF (echo) (%)	57 ± 10	58 ± 11	*p* = 0.88
IVS thickness (echo) (mm)	13 ± 3	15 ± 2	*p* = 0.33
I-LV EDV (CMR) (ml/m^2^)	61 ± 13	73 ± 27	*p* = 0.10
LV EF (CMR) (%)	58 ± 15	55 ± 10	*p* = 0.15
I-LVM (CMR) (g/m^2^)	115 ± 60	113 ± 30	*p* = 0.38
I-RV EDV (CMR) (ml)	58 ± 13	58 ± 13	*p* = 0.90
Blood/Myocardial T1 ratio	0.6 ± 0.08	6.2 ± 1.8	*p* < 0.001
RV EF (CMR) (%)	58 ± 14	58 ± 11	*p* = 0.30
Serum creatinine (mg/dl)	1.6 ± 1.2	1.2 ± 0.6	*p* = 0.17
Hematocrit (%)	45 ± 5	38 ± 6	*p* = 0.45
Hemoglobin (g/dl)	14.2 ± 0.9	13.8 ± 0.9	*p* = 0.39
History of hypertension (*n*)	5	3	*p* = 0.99
Smoke habit (*n*)	3	2	*p* = 0.99
Diabetes (*n*)	3	3	*p* =1

*CMR* cardiac magnetic resonance, *LV EDV* left ventricular end diastolic volume, *LV EF* left ventricular ejection fraction, *IVS* interventricular septum, *I-LV EDV* indexed left ventricular end diastolic volume, *I-LVM* indexed left ventricular mass, *I-RV EDV* indexed right ventricular end diastolic volume, *RV EF* right ventricular ejection fraction

**Table 3:** Univariate and multivariate analysis

Variabile	Univariate HR (95% CI)	*p*-value	Multivariate HR (95% CI)	*p*-value
LVEDV MRI (10 ml step)	0.99 (0.96–1.03)	0.22		
RVEDV MRI (10 ml step)	**1.02 (1.01–1.03)**	**0.03**	1.01 (0.96–1.02)	0.12
I-LVM MRI (10 g/m^2^ steps)	0.98 (0.97–1.01)	0.97		
LV EF MRI (5-point step)	**0.89 (0.80–0.99)**	**0.04**	0.99 (0.98–1.01)	0.20
RV EF MRI (5-point step)	0.98 (0.97–0.99)	0.03		
Age	**1.03 (1.01–1.11)**	**0.04**	1.01 (0.96–1.11)	0.15
E/e’	1.01 (0.96–1.11)	0.55		
Serum Creatinine (0.5 mg/dl steps)	1.2 (0.71–1.71)	0.59		
Typical amyloidosis (yes)	**3.6 (1.2–8.8)**	**<0.01**	**3.2 (1.5–6.4)**	**<0.01**

*LVEDV* left ventricular end diastolic volume, *RVEDV* right ventricular end diastolic volume, *I-LVM* indexed left ventricular mass, *LV EF* left ventricular ejection fraction, *RV EF* right ventricular ejection fraction, *MRI* magnetic resonance imaging

## Discussion

CMR is the gold standard for structural and functional evaluation of patients with known or suspected cardiomyopathy. The prognostic value of CMR has been documented in ischaemic [6], dilated [7] and hypertrophic cardiomyopathy [8, 9]. In CA, the evidence of global subendocardial LGE with accelerated contrast washout has shown an excellent diagnostic accuracy [10].

Data from our study suggest that the presence of typical LGE patterns is a strong independent predictor of mortality for patients with suspected CA. Currently, little evidence is available about the role of LGE as predictor of adverse events in CA. Maceira et al. didn’t find any significant correlation between LGE patterns and survival at 4 years in 29 patients with proven AL CA, while a correlation was found for gadolinium kinetics [11]. Although a trend towards a worse outcome was observed for patients with positive LGE, the sample size was probably too small to draw any definitive conclusion. The larger prospective study by White et al. documented the prognostic value of transmural LGE in a cohort of patients with proven systemic amyloidosis and suspected cardiac involvement [12]. Considering that CA is a progressive disease and the degree of LGE probably reflects the continuum of myocardial involvement, the former study mainly identified patients with advanced disease featuring an intrinsic worse prognosis. This observation agrees with the findings by Fontana et al. [13]. who demonstrated that transmural global LGE was a stronger death predictor than subendocardial LGE. In our population, such prognostic difference can’t be observed because of the relatively small quota of transmural LGE. Recently, a large meta-analysis of 425 patients demonstrated an increased risk of death after a median follow-up of 25 months in patients with positive LGE (odds ratio 4.96, 95% CI 1.9–12.9) [14]. However, heterogeneity of inclusion criteria, imaging protocols and follow-up length within the studies could have determined a loss of events, especially for patients with early stage disease. To the best of our knowledge, the present study has the longest follow-up available for this category of patients. Our data show that the median survival time is significantly shorter for patients with a typical LGE pattern for CA. Conversely, the absence of a typical LGE was associated with better long-term prognosis, independently from typical predictors of worse outcome for heart failure patients like left ventricular ejection fraction or E/e’ (as a surrogate of diastolic dysfunction) [15]. I‑LVM, often considered a marker of cardiac involvement in infiltrative diseases [16], did not show any adjunctive predictive role compared to LGE. Our data, derived from a “real world” scenario, suggest that the LGE pattern can be considered a reliable tool for the diagnosis of CA. Moreover, the degree of myocardial involvement may play a central role in prognostic stratification of patients with suspected CA. In our population, the mere presence of LGE in the heart was associated with a significantly worse outcome. This probably accounts for the possibility that myocardial involvement could be one of the last steps of a progressive systemic disease. However, over the course of the past few years, new therapeutic approaches have been developed for targeted therapy of most forms of CA [17]. Although there still is no solid data about the efficacy of such interventions on patients with heavy myocardial involvement, an adjunctive tool for prompt identification of high-risk patients could accelerate the planning of further invasive and therapeutic strategies. On the other hand, if these new therapies do not prove to be effective in this subgroup of patients, severe myocardial involvement at CMR could reasonably be considered an exclusion criterion for the administration of such treatments.

Some limitations of the present study should be acknowledged. This is a retrospective study carried out in a tertiary referral centre, thus with a high probability of including patients with severe disease. In recent years, the introduction of parametric mapping has been shown to be very promising in patients affected by infiltrative disorders, allowing a better characterisation of the disease and an early diagnosis. At the time of the study, T1 mapping was not yet available at our centre, so it could not be included in the scan protocol of these patients. Besides, T1 mapping is not yet completely standardised across centres, while LGE acquisition and assessment are well established and are part of routine CMR clinical examination, thus rendering the analysis of LGE pattern an easily obtainable tool for the clinical evaluation of patients with CA. T1 blood/myocardial ratio is not a validated technique for the assessment of contrast washout and inversion times were chosen at the operators’ discretion during acquisition of LGE sequences. However, this method is a physiologically logical surrogate for semi-quantitative description of contrast washout and showed good correlation with inspective evaluation of gadolinium kinetics. Another significant limitation of the study is the exclusion of patients with severe renal failure due to contraindication to a magnetic resonance contrast agent. Considering that most forms of systemic amyloidosis show a progressive renal involvement, this limitation could result in the exclusion of some patients with an advanced stage disease, potentially leading to a selection bias.

In conclusion, the LGE pattern proved to be an affordable diagnostic tool and an independent adverse prognostic predictor for patients with suspected cardiac amyloidosis. Early identification of high-risk subjects could guide towards a more aggressive diagnostic workup, helping to improve therapeutic choices.
